# Immersive competence as a source of bias in virtual reality clinical assessment

**DOI:** 10.1038/s41746-026-02482-z

**Published:** 2026-03-09

**Authors:** Jan Schaal, Tobias Leutritz, Marco Lindner, Alexander Zamzow, Joy Backhaus, Sarah König, Tobias Mühling

**Affiliations:** University Hospital Würzburg, Institute of Medical Teaching and Medical Education Research, Würzburg, Germany

**Keywords:** Health care, Medical research, Neuroscience, Psychology, Psychology

## Abstract

Virtual reality (VR) is increasingly used for assessment in educational and clinical settings. However, users’ immersive competence (IC)—the ability to navigate and operate VR systems—may introduce bias unrelated to clinical skills or patient functioning. In this randomized controlled trial, 88 medical students received either general IC training, general+specific IC training, or no structured training before completing a VR-based assessment scenario. Multimodal data were collected, including electrodermal activity, cognitive-load ratings, procedural efficiency, and usability barriers. Specific IC training improved performance compared with control (28.3% ± 10.3% vs. 21.2% ± 10.8%, *p* = 0.010, *d* = 0.67), moderated by procedural efficiency and increased cognitive load. Prior 3D experience did not predict performance in the control group, likely due to a floor effect, but did in the specific training group. These findings indicate that IC is a causal, modifiable factor in VR-based assessments and should be considered to ensure fair and valid evaluations.

## Introduction

Virtual Reality (VR) is increasingly used not only in medical education^1^ but also in clinical diagnostics and therapy^2^. In immersive environments, users can engage in realistic and standardized simulations to practice or assess professional skills^1,3^, monitor cognitive and motor functions^4–6^, or receive psychological or rehabilitative interventions^7–11^. However, as these applications gain momentum in high-stakes contexts, questions of validity and equitable access become critical. VR systems are neither standardized nor always intuitive, leading to substantial variations in functionality and usability^12,13^. Learners and patients alike must master abstract interaction metaphors, interpret spatial feedback, and operate handheld controllers^12–15^. This creates a hidden source of variance, where prior informal digital experience (e.g., video gaming) can unfairly advantage certain users^16–19^. Yet, the underlying ability responsible for this effect has remained largely underdefined.

Recent work has introduced the concept of Immersive Competence (IC)—the user’s familiarity and skill in operating VR systems^17,18,20^—as a potentially construct-irrelevant factor^21^ influencing performance. From a psychometric perspective, this threatens the validity of VR-based assessments, particularly the extrapolation inference^22^, by conflating technological fluency with domain-specific ability (e.g., clinical performance). Despite this, none of the 26 VR-based assessments in health professions education listed in a recent systematic review accounted for IC as a potential confounder^3^. Likewise, clinical VR assessments in neurology^4,23^, psychiatry^5,6,9,24^, or geriatrics^25^ have almost exclusively focused on task outcomes, while largely overlooking patients’ ability to operate these immersive systems. However, without accounting for IC, VR-based evaluations risk perpetuating unseen performance biases by disadvantaging less digitally experienced learners or patients and undermining fairness and validity.

To address this gap, recent tools have been developed to measure and train IC in a standardized manner, covering general interaction categories such as manipulation, navigation, and system control^17,18^ and to train and assess domain-specific IC directly within a target VR environment^18^. Yet, empirical studies demonstrating whether IC causally influences clinical performance—and whether IC training can uniformly mitigate performance differences—remain lacking. Importantly, training effects may follow complex, dose- and baseline-dependent trajectories: cognitive training can produce either compensation, where lower‑skill individuals benefit most, or magnification, where those with greater initial resources gain even more^26,27^. Moreover, recent evidence indicates that the direction and magnitude of these effects depend on the cognitive domain, the training format (e.g., process- vs. strategy-based), and individual characteristics such as age^26,28^.

Against this background, the present study investigates whether IC training systematically affects performance in a VR-based clinical assessment. We hypothesized that (H1) targeted IC training would enhance clinical performance compared to no training, (H2) this effect would be moderated by increases in procedural efficiency (H2a) and/or reductions in cognitive load (H2b), (H3) IC training would mitigate performance disparities associated with prior 3D or VR application experience, and (H4) trained participants would report less distraction by usability barriers and a more positive evaluation of the VR-based assessment. Findings aim to inform the design of equitable and valid VR-based tools in both education and healthcare—ensuring that performance reflects actual clinical competence in examinees and functional ability in patients rather than technological familiarity.

## Results

### Participant characteristics

**Fig. 1:**
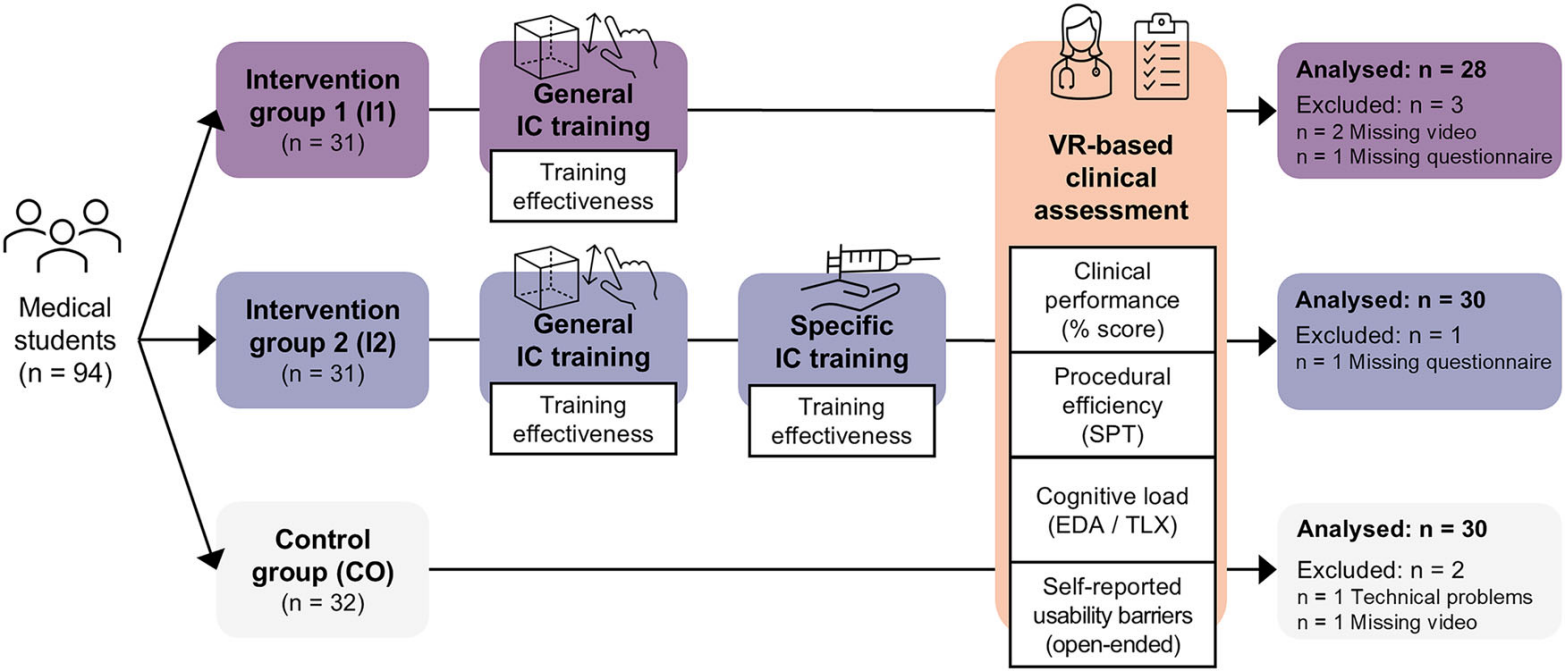
Study design and data collection process for IC training and VR-based assessment of clinical performance. EDA electrodermal activity, IC immersive competence, TLX NASA task load index, SPT standardized procedural time, VR virtual reality.

A total of 94 medical students were enrolled and randomized across the three study arms (Fig. 1). Due to technical problems with the VR hardware/software, missing video recordings and incomplete questionnaires, six datasets were excluded from the final analysis (I1: *n* = 3, I2: *n* = 1, CO: *n* = 2). The remaining 88 participants were representative of the overall population of advanced undergraduate medical students (Table 1). Academic performance, measured as the exam score, was slightly better in CO compared to I1 and I2 (*p* = 0.020). Overall, participants reported limited prior exposure to 3D applications (mean: 9.36 ± 37.0 h in the past 6 months) and minimal prior VR experience (mean: 0.07 ± 0.23 h in the past 6 months), consistent with findings from comparable cohorts in medical education.

**Table 1:** Participant characteristics and experiences across groups

Characteristics and experiences		Total (*n* = 88)	I1 (*n* = 28)	I2 (*n* = 30)	CO (*n* = 30)	*p* value
Gender, *n* (%):	Female Male Diverse	63 (72%) 25 (28%) 0 (0%)	18 (64%) 10 (36%) 0 (0%)	22 (73%)8 (27%) 0 (0%)	23 (77%) 7 (23%) 0 (0%)	0.56
Age (years), mean ± SD		24.5 ± 2.8	25.0 ± 3.6	24.9 ± 2.6	23.6 ± 2.0	0.169
Exam score^a^, mean ± SD		2.14 ± 0.73	2.34 ± 0.83	2.29 ± 0.66	1.83 ± 0.60	**0.020**
3D experience (hours)^b^, mean ± SD		9.36 ± 37.0	12.0 ± 31.6	12.2 ± 54.8	4.0 ± 11.6	0.630
VR experience (hours)^b^, mean ± SD		0.07 ± 0.23	0.11 ± 0.28	0.05 ± 0.20	0.07 ± 0.22	0.641

German grading system: 1 = best, 5 = worst.

^a^Exam score = mean grade across internal medicine, emergency medicine, and anesthesiology.

^b^Refers to the past 6 months.

### General and specific IC training effectiveness

**Fig. 2:**
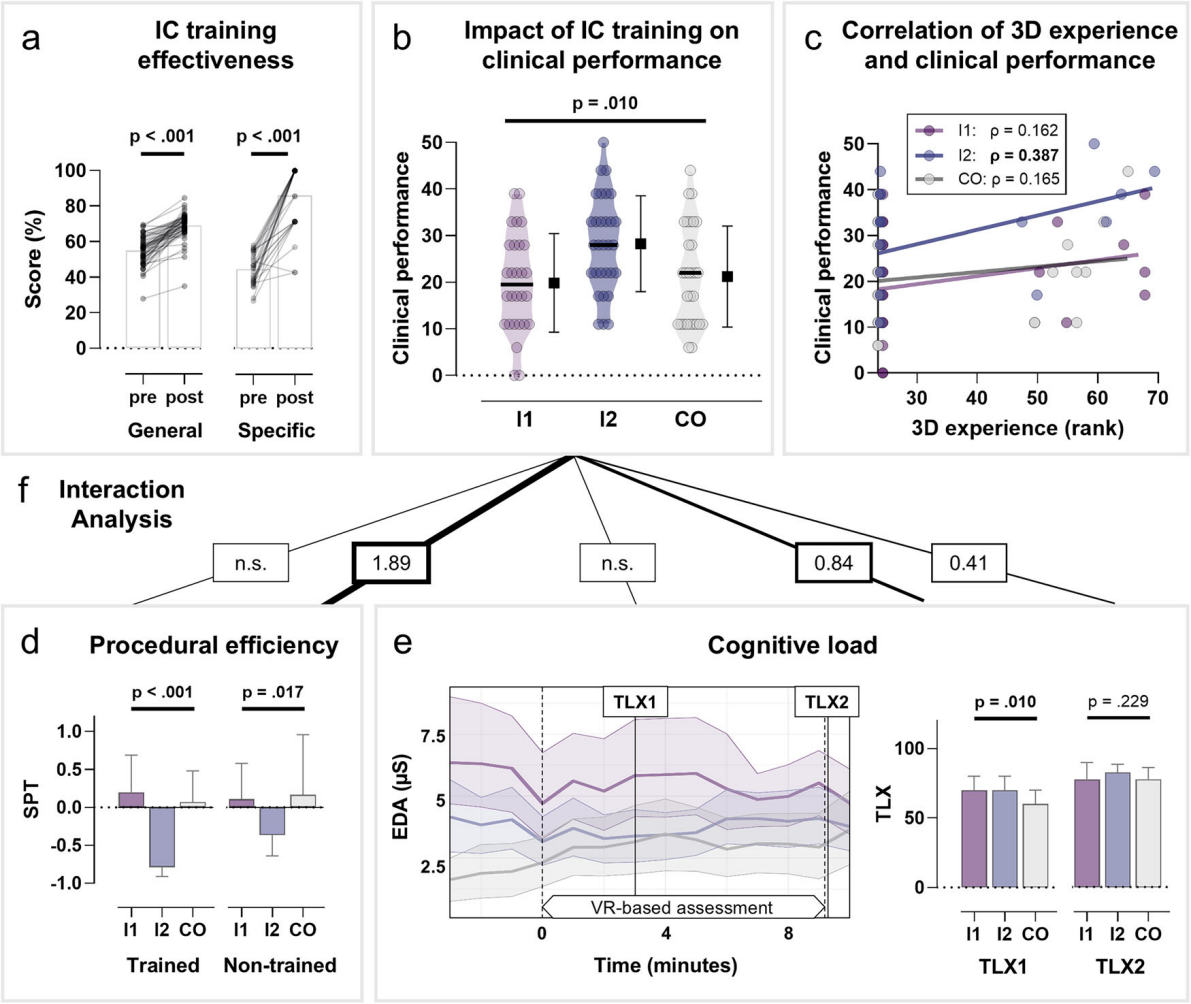
Primary and secondary outcomes. **a** Effectiveness of general and specific IC training. Bar graphs depict group means, with overlaid dots representing individual participants in the pre–post comparison. **b** Impact of IC on clinical performance in the VR assessment, illustrated by violin plots with medians (black horizontal lines) and means ± SD (square symbols with error bars). **c** Correlation between 3D experience (expressed as rank) and clinical performance across groups. Individual data points are shown together with their respective regression lines. Ranks reflect the relative position of each participant’s 3D experience within the combined sample. **d** Procedural efficiency comparing previously trained (left) and untrained (right) measures. **e** Cognitive load, including progression of EDA during VR assessment and group differences for NASA-TLX scores at time points 1 (during assessment) and 2 (immediately after assessment). **f** Effect size of the interaction analysis: influence of IC on clinical performance through cognitive load and procedural efficiency. The values inside the boxes represent Cohen’s *d*. CO control group, EDA electrodermal activity, I1/I2 intervention group 1/2, IC immersive competence, SPT standardized procedural time, TLX task load index, VR virtual reality.

Both general and specific training modalities resulted in significant gains of IC (Fig. 2a). Participants who received general IC training (I1 and I2) improved their general IC score from 54.9% to 69.1% (*p* < 0.001), mean training time 24.1 ± 2.3 min. Participants in the specific IC training group (I2) increased their specific IC score from 44.7% to 86.2% (*p* < 0.001), mean training time 21.7 ± 3.3 min.

### Impact of IC training on clinical performance (H1)

As the primary outcome, clinical performance in the VR-based emergency scenario differed significantly between groups (Fig. 2b). Mean performance scores were 19.9% ± 10.6% in I1 (general training), 28.3% ± 10.3% in I2 (general + specific training), and 21.2% ± 10.8% in CO. While performance in I1 did not substantially differ from CO, participants in I2 achieved markedly higher scores than both other groups. This pattern was confirmed by Kruskal–Wallis test (*p* = 0.010) and post hoc analyses, with both unadjusted and FDR-adjusted p-values (Supplementary Table 4) indicating that the combined training condition (I2) was the only intervention associated with a clear performance advantage corresponding to a medium-to-large effect size (Cohen’s *d* = 0.67) compared to CO.

Given the unequal distribution of exam scores across groups, we controlled for this variable using ANCOVA, after which the significant group effect on clinical performance remained virtually unchanged (Cohen’s *d* = 0.64). Interestingly, exam score and clinical performance were not correlated (*r* = 0.14, *p* = 0.24). Interrater reliability of checklist scoring was consistently high, with a linear weighted Cohen κ of 0.895 across all items.

### Procedural efficiency as a moderator (H2a)

To evaluate the effect of training on procedural efficiency, SPT was analyzed for one trained procedure (intravenous cannulation) and four untrained transfer tasks (blood culture sampling, temperature measurement, abdominal ultrasound, pneumatic tube operation) (Fig. 2d). For the trained procedure, mean SPT differed significantly between groups (Kruskal–Wallis: *p* < 0.001) accompanied by a large effect size (Cohen’s *d* = 1.59). When controlling for exam scores Cohen’s *d* dropped slightly to 1.19. Participants in I2 demonstrated the shortest SPT (−0.683 ± 0.332), followed by CO (0.260 ± 0.853) and group I1 (0.443 ± 1.181). For the aggregate SPT of the untrained transfer tasks, group differences were less pronounced but still significant (Kruskal–Wallis: *p* = 0.017), which corresponds to a moderate value of Cohen’s *d* (0.56). When controlling for exam scores Cohen’s *d* increased markedly to a large value of 0.93. SPT was −0.368 ± 0.424 in I2, 0.184 ± 0.662 in I1, and 0.214 ± 0.806 in CO. Across groups, SPT for untrained tasks showed a significant main effect on clinical performance (*p* < 0.01, Cohen’s *d* = 1.15), whereas SPT for trained tasks did not.

Students with low SPT values (greater efficiency) in untrained tasks achieved higher clinical performance (Cohen’s *d* = 1.89). Accordingly, SPT functioned as a moderating variable. Expressed as a regression equation, clinical performance decreased on average by *Y* = 32.6 **−** 8.70 for low values of untrained SPT and by *Y* = 32.6 − 17.3 for high values. Thus, high SPT values (indicating low efficiency) in untrained tasks were associated with approximately a 50% reduction in clinical performance.

### Cognitive load as a moderator (H2b)

Objective cognitive load, assessed via EDA, did not differ significantly across groups during the VR assessment (Fig. 2e, left). While there were group differences immediately before the assessment (minutes −3 to −1) with I1 showing the highest median EDA levels, followed by I2 and CO (Kruskal-Wallis: all *p* < 0.002), median EDA values converged during the assessment (minutes 0–10). Transient differences at isolated time points did not remain significant after correction for multiple testing (Kruskal-Wallis after FDR adjustment, Supplementary Table 6). However, a trend toward higher EDA in I1 compared to the other groups persisted throughout the assessment period. Importantly, exploratory baseline-normalized analyses resulted in artificial distortions due to substantial between-group variability in baseline values and were therefore not considered further.

During the VR assessment (TLX1), subjective NASA-TLX ratings showed a small but significant group difference. Surprisingly, CO reported the lowest subjective cognitive load (I1: 69.4 ± 18.9, I2: 68.0 ± 15.6, CO: 58.6 ± 15.6; Kruskal–Wallis: *p* = 0.010). Immediately after the assessment (TLX2), however, subjective cognitive load was similar across groups (I1: 75.8 ± 17.8, I2: 80.9 ± 13.9, CO: 77.3 ± 10.6; Kruskal–Wallis: *p* = 0.229) (Fig. 2e, right).

Within-group correlations between subjective cognitive load and procedural efficiency were largely negligible in the CO group at both timepoints, for both trained (*r* = −0.04, *p* = 0.88 at TLX1; *r* = 0.13, *p* = 0.54 at TLX2) and untrained tasks (*r* = −0.04, *p* = 0.88 at TLX1; *r* = 0.05, *p* = 0.87 at TLX2). In the I2 group, a moderate, non-significant negative correlation emerged for the trained task (*r* = −0.23, *p* = 0.22 at TLX1; *r* = −0.35, *p* = 0.06 at TLX2), linking higher load to shorter SPTs (greater efficiency). The untrained task remained largely unrelated at TLX1 (*r* = 0.03, *p* = 0.90) and also showed a negative trend at TLX2 (*r* = −0.25, *p* = 0.30).

EDA did not show significant moderating effects between groups and clinical performance. In contrast, subjective ratings of cognitive load (TLX1) demonstrated a strong main effect on performance (*p* < 0.001), irrespective of group membership. Students with medium NASA-TLX values achieved the best clinical performance, followed by those with high values (Cohen’s *d* = 0.84). Expressed as regression equations, clinical performance increased by *Y* = 7.80 + 18.8 for medium NASA-TLX values and by *Y* = 7.80 + 15.1 for high values. A similar, though non-significant pattern was observed for the TLX2 when comparing I2 to CO. Again, students with medium NASA-TLX values showed the highest performance, followed by those with high values (Cohen’s *d* = 0.41, regression equation: medium *Y* = 11 + 16.0, high: *Y* = 11 + 13.9).

### Correlation between 3D experience and clinical performance (H3)

Values of prior 3D experience were not normally distributed across groups (Shapiro-Wilk test, all *p* < 0.001) and overall very low, with 75% of participants reporting a value of 0, resulting in severely restricted score ranges that limit the interpretability of correlation analyses. Spearman correlations indicated small, non-significant associations in CO (*ρ* = 0.165, *p* = 0.383) and I1 (*ρ* = 0.162, *p* = 0.409), but a moderate, significant correlation in I2 (*ρ* = 0.387, *p* = 0.034) (Fig. 2c). As the participants had virtually no prior VR experience (Table 1), no correlation with clinical performance was observed for this variable (all *ρ* < 0.100).

### Self-reported usability barriers and acceptance (H4)

Across all groups, enjoyment ratings for the VR scenarios were high (I1 = 4.39 ± 0.69, I2 = 4.53 ± 0.63, CO = 4.14 ± 0.87; Kruskal–Wallis *p* = 0.725), and perception of future applicability of VR-based examinations was predominantly positive (I1 = 3.11 ± 1.13, I2 = 3.57 ± 1.07, CO = 3.21 ± 1.24; Kruskal–Wallis: *p* = 0.284). However, participants in I1 and CO reported significantly more distraction from clinical content due to interface barriers compared to I2 (I1 = 3.00 ± 1.25, I2 = 2.47 ± 1.17, CO = 3.38 ± 1.37; Kruskal–Wallis: *p* = 0.024). Spearman correlation analyses revealed a significant negative association between prior 3D experience and distraction scores in I2 (*ρ* =  0.433, *p* = 0.017), whereas no significant association was observed in CO (ρ = −0.196, p = 0.309).

A total of 91 responses to the open-ended question on prerequisites for future acceptance of VR-based examinations were coded for thematic analysis (Supplementary Table 3). Three main priorities emerged: (1) Most participants emphasized the need for prior preparation with the VR environment, either theoretically (23/91, 25%) or practically (66/91, 73%), with 78% mentioning at least one of these forms and some additionally suggesting early curricular integration (16/91, 18%). Extensive training was viewed as essential to avoid inequities in operating the simulation. (2) A considerable number suggested program-related improvements to enhance accessibility (17/91, 19%), such as increased fonts or facilitated grasping interactions. (3) Finally, several participants also recommended adjustments to the examination conditions (11/91, 12%), such as extending the time limit or modifying evaluation criteria.

## Discussion

This randomized controlled trial provides the first causal evidence that IC—defined as a user’s ability to navigate and interact with VR systems—significantly influences clinical performance in VR-based assessments (confirming H1). Participants who received specific IC training achieved higher scores than untrained peers in a complex, time-sensitive medical simulation. This effect remained robust after adjusting for baseline academic exam scores, demonstrating that the observed performance advantage was not attributable to pre-existing academic differences. From a validity perspective, IC represents construct-irrelevant variance that may compromise the extrapolation inference in assessment design^21,22^. Analogous to challenges encountered in early computer-based testing^29,30^, our data indicate that VR-based performance metrics may conflate true clinical competence with interface proficiency. Interestingly, training general interaction techniques alone was insufficient—instead, context-specific training within the target application (specific IC training) proved necessary to achieve measurable performance benefits.

Importantly, our findings extend beyond education. Comparable dynamics are likely in clinical VR applications—such as diagnostics in neurology, psychiatry or geriatrics^5,6,23–25^—where patients with limited digital abilities may underperform for non-clinical reasons, leading to misclassification or inappropriate therapeutic decisions. Such disparities mirror second-order digital divides in digital health, where user capability—rather than hardware access—limits participation^31–33^. Especially patient populations with little exposure to digital tools, including older adults, people with disabilities, and socioeconomically disadvantaged groups, may therefore experience recurrent disadvantages in VR-based diagnostics and interventions. While such issues are already well recognized in the context of algorithmic bias research^34,35^, they remain underexplored in immersive systems for educational and clinical assessments^8,10^.

Procedural efficiency, measured as SPT, emerged as a plausible moderator (confirming H2a). Group effects appeared for both trained and untrained tasks, with I2 participants completing more clinically relevant actions within the limited timeframe, which is consistent with effects from other VR trainings^36,37^. Although some basic interaction principles from specific IC training were conceptually similar to steps encountered in the assessment, no training task directly mirrored the assessment items. Interestingly, only SPT for untrained tasks contributed significantly as a moderator. Thus, clinical outcomes depended less on executing rehearsed steps quickly than on managing novel scenario components efficiently. Notably, SPT in untrained tasks was a strong predictor of clinical performance even after correction for baseline knowledge (exam score), supporting a role as a distinct component of immersive competence.

Similar to clinical performance, general IC training (I1) had little effect on SPT, likely due to the higher cognitive and motor demands of high-fidelity VR environments^13,38^, for which general engagement with VR interaction techniques was likely insufficient. This limited impact may reflect differences in controller mappings and graphical representations between general IC training and assessment, as well as the fact that this training type - unlike the specific training and the assessment - focused on isolated interaction patterns rather than integrated task sequences. Comparable patterns have been documented in studies on internet skills and motor tasks, where general abilities show limited transfer to strategic or context-specific applications^39,40^. Altogether, these findings highlight procedural efficiency as both a sensitive proximal outcome and a potential mechanism for broader skill transfer in immersive simulations^41,42^.

Although cognitive load was also hypothesized as an interacting variable, the results were unexpected. Both NASA-TLX and EDA indicated lower load in the CO, yet these participants achieved the weakest clinical outcomes, and no linear moderating role was observed (refuting H2b). Added to that, subjective cognitive load showed an interaction effect, with medium values yielding the best performance^43^. Taken together, these observations challenge the assumption that IC training primarily reduces extraneous cognitive burden^44^, at least when training and assessment occur back-to-back. One explanation is that training itself induced substantial activation, creating a carry-over effect masking downstream reductions in cognitive load^45^. The high pre-assessment EDA in I1, who trained under the greatest time pressure due to rapid task repetition, supports this interpretation. Since this effect also appeared to influence the earlier baseline measurement between minute −6 and −4, we therefore reported native, non–baseline-corrected EDA values. However, cognitive load theory offers an additional explanation^46^. Low TLX1 ratings in CO might also reflect disengagement with task-relevant actions due to difficulties in VR interaction. In contrast, the higher load reported by trained participants might have been primarily germane—arising from active problem solving and procedural engagement^47,48^—and thus conducive to superior performance. This interpretation is supported by within-group correlations, which suggested that in CO, subjective load was largely unrelated to procedural efficiency, whereas in I2 higher load tended to co-occur with greater efficiency (lower SPT), in line with the conceptualization of germane load as a redistribution of cognitive resources toward task-relevant processing^49^. Although these correlations were not statistically significant and effect sizes were moderate, they are compatible with the idea of non-productive versus productive cognitive load.

Although we assessed cognitive load not only retrospectively, but also during the simulation using multiple measures^50^, especially EDA, may lack the specificity required to disentangle cognitive from emotional arousal^51,52^. Thus, more precise methods (e.g., eye-tracking)—which were not available in combination with VR headsets at the time of the study—might provide a more detailed picture in the future.

Non-parametric correlation analyses revealed a pattern opposite to our initial hypothesis that IC training would mitigate digital advantages (refuting H3). Unlike previous studies^16,18,19^, in our population, unexposed to IC training (CO), no relevant association between prior 3D experience and clinical performance was detectable. In contrast, after specific training (I2), higher levels of prior 3D exposure were moderately related to superior performance, just exceeding the significance level. However, participants reported uniformly low 3D experience (mean <10 h over the past 6 months, with 75% reporting zero), resulting in a floor effect with limited variance. Such restricted score ranges are known to attenuate rank-based coefficients like Spearman’s *ρ*, which depend on sufficient dispersion in both variables to detect monotonic relationships^53^. In light of this, a correlation between prior 3D exposure and clinical performance in CO may still exist, even if it was mathematically undetectable, justifying a very cautious interpretation of the results related to H3.

However, if the observations reflect a genuine effect, IC training may not function solely as an equalizer but rather as a conditional facilitator that allows participants to leverage pre-existing competencies once basic interaction barriers are removed. This dynamic parallels the “Matthew effect” (magnification theory) described in cognitive^27^ and educational research^54,55^, where training may amplify rather than level initial differences. One contributing factor may be the relatively short, 25-min training duration during which participants may not have reached a leveling threshold—the point at which additional practice enables lower-skilled individuals to catch up with higher-skilled peers^27^. Early training may thus accentuate initial differences, whereas compensatory effects typically emerge only after sufficient intensity or duration^27,54^, and are further moderated by individual baseline abilities and cognitive characteristics^26^. This also aligns with dose-response studies indicating that training benefits follow nonlinear trajectories, with extended or repeated sessions required to offset baseline disparities^28^. Future work should systematically vary training duration or frequency to determine whether longer IC training produces more robust compensatory effects in digitally inexperienced learners.

High enjoyment ratings and generally positive outlook on the fairness of VR-based examinations align with prior reports that immersive technologies are well accepted in medical education when they are perceived as engaging and safe learning environments^56^, with a strong sense of presence and place further supporting positive emotional and motivational responses in users^57^. At the same time, the pronounced distraction caused by interface barriers in untrained participants (confirming H4) reflects well-documented usability challenges of VR systems, including navigation difficulties, limited haptic feedback, and inconsistent interaction metaphors^12,13,58^. Similar to H3, a potential facilitator effect was also observed for subjective distraction, as correlations with prior 3D experience only emerged after specific IC training. The training may have helped participants with prior 3D experience recognize familiar interaction patterns in VR, potentially reducing perceived distraction (extraneous cognitive load)^49,59^. However, these correlations should be interpreted cautiously, given the skewed distribution of prior 3D experience.

**Fig. 3:**
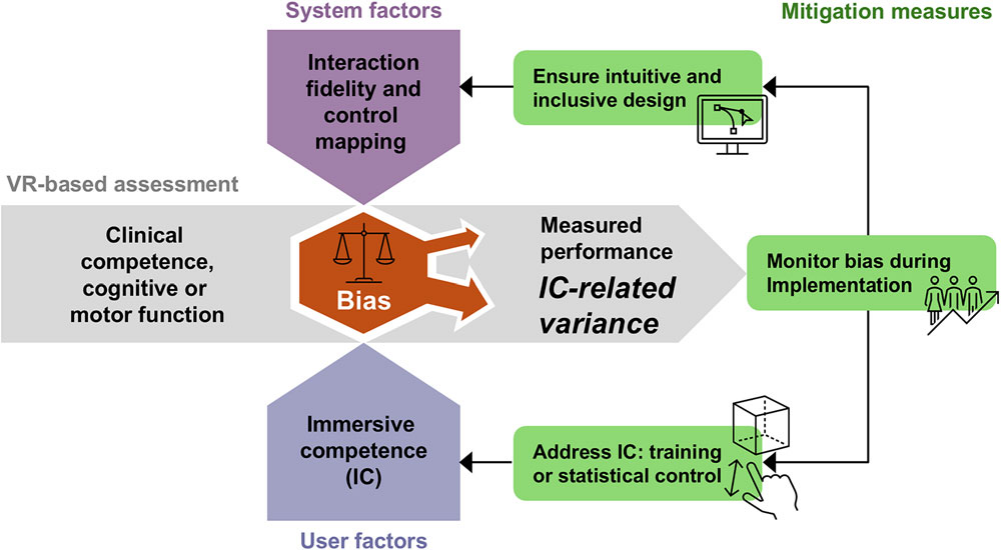
Conceptual model of IC bias in VR-based assessments and potential mitigation strategies. System factors (e.g., low interaction fidelity, unfavorable control mapping) and user factors (varying levels of IC) may introduce bias into VR-based clinical or functional assessments. In the main assessment flow (horizontal arrow), the intended target constructs—clinical competence and cognitive/motor function—become contaminated by IC-related variance. Mitigation measures are shown on the right: intuitive and inclusive design addresses system factors, while IC training or statistical control addresses user factors. Monitoring performance bias during implementation feeds back into both domains, enabling continuous detection and reduction of bias. IC immersive competence, VR virtual reality.

The current study demonstrates that failure to account for IC may reinforce digital inequities while creating a misleading appearance of objectivity. To address this risk, we propose three actionable mitigation strategies (Fig. 3):

1. Design for inclusive interaction: Developers should adhere to principles of intuitive interface design—reducing unnecessary complexity, standardizing interaction metaphors, and applying universal design elements^56,60^. These measures help minimize dependence on IC and enable participation by users with diverse digital backgrounds.
2. Monitor for bias during implementation: Performance data should be continuously monitored for correlations with digital background characteristics. Any unexpected disparities may indicate hidden dependencies on IC and require adjustments of the tool or its deployment protocol.
3. Address IC through training or statistical control: Before implementing immersive assessments, it is important to clarify how differences in IC will be addressed. Where feasible, standardized IC training modules can be provided, while monitoring their potential to amplify pre-existing advantages. When training is not feasible or its effects are uncertain, IC should be measured and accounted for in analyses—either as a covariate or as a stratification factor.

Future research should first clarify the role of IC training. It remains uncertain whether training primarily reduces disparities or amplifies pre-existing advantages, and which doses, formats, or contexts promote levelling effects. Identifying reliable training protocols is therefore central to ensuring fair educational and clinical use. Longitudinal studies are also needed to trace how IC develops across educational stages and patient populations, and whether its influence on performance endures over time, particularly as extended reality becomes part of daily life. In parallel, cross-cultural and demographic research should examine how socioeconomic status, age, and technology access shape IC, linking it to broader issues of digital health equity. Finally, studies must determine how IC biases diagnostic conclusions in clinical domains such as neurology, psychiatry, and geriatrics, and how these biases can be mitigated through design improvements, targeted training, or calibration strategies.

This study has several limitations. First, the sample consisted of medical students from a single German institution, which may restrict the generalizability of findings to other educational or clinical populations. Digital fluency and prior exposure to immersive systems likely vary across regions, age groups, and socioeconomic backgrounds.

Second, our study tested a single, relatively brief IC training protocol. It therefore remains unclear whether different training doses, formats, or contexts might yield distinct effects—for example, acting as levelers in some cases and amplifiers in others. This uncertainty limits the generalizability of our findings and highlights the need for systematic variation of IC training designs in future work.

Third, due to the extremely low and uniform levels of prior 3D experience in our sample (75% reporting 0 h), the resulting floor effect limits the variance available to detect systematic associations, constraining causal claims regarding H3 and subjective distraction (H4). These findings should therefore be considered exploratory.

Fourth, the measures of cognitive load have inherent constraints: EDA lacks specificity to distinguish cognitive from emotional or physical arousal, while NASA-TLX is subjective and susceptible to individual response tendencies. In addition, the EDA baseline was recorded while participants were already immersed in the VR environment and likely reflected anticipatory or carry-over effects from prior training rather than a true resting state. As a result, baseline-normalized EDA analyses were not interpretable, and only native EDA values were reported, which should be considered when interpreting group differences in autonomic arousal.

Finally, the VR scenarios focused exclusively on internal medicine emergencies. Although valid for this context, the findings may not be directly transferable to other specialties or patient populations.

To conclude, immersive competence may be a critical yet overlooked determinant of user performance in VR-based healthcare applications. Our findings demonstrate that targeted IC training can significantly enhance medical task execution and may help mitigate performance disparities, although its effects may depend on baseline user abilities and training conditions.

Without accounting for IC, immersive technologies risk introducing unintended bias, compromising both the fairness and validity of high-stakes assessments and digital interventions. As VR continues to expand into clinical diagnostics, rehabilitation, and professional education, systematic strategies for measuring, training, or mitigating IC will be essential. Recognizing immersive competence as a modifiable, equity-relevant factor can help ensure that VR-based systems serve as enablers—rather than barriers—in advancing accessible and just digital health.

## Methods

### Study design and setting

This single-center, randomized controlled trial was conducted between January and August 2025 at the clinical skills laboratory of a German medical faculty. The aim was to test whether IC training influences performance in a VR-based clinical assessment. Eligible participants were advanced undergraduate medical students who had completed the internal medicine written exam (end of 7th semester). Exclusion criteria were known epilepsy or severe simulator sickness. Recruitment occurred via semester mailing lists, and enrollment was online. Sessions took place individually, outside curricular teaching hours, in a standardized VR laboratory. Participants received a €25 book voucher upon study completion.

Participants were randomly allocated (1:1:1) to three groups using a computer-generated sequence with a fixed block size (*n* = 9). Due to visible differences in training, blinding for participants was not feasible. However, performance was recorded automatically and rated from anonymized videos, ensuring rater blinding (single-blind design).

Power analysis (one-way ANOVA, *α* = 0.05, power = 0.80) indicated that a minimum of 84 participants would be needed to detect a moderate-to-large effect size (*f* = 0.25). To account for potential attrition, 94 were enrolled.

### Interventions

Participants were assigned to one of three study groups:

- Intervention group 1 (I1): general IC training (abstract VR interactions).
- Intervention group 2 (I2): general + specific IC training (in-examination environment).
- Control group (CO): no structured IC training.

After providing informed consent and a basic familiarization with VR controls, I1 and I2 completed their respective IC training sessions. All groups then underwent the same VR-based clinical assessment.

General IC training was delivered using the VR Competence App^17^, which includes eight modular interaction challenges (e.g., object manipulation, navigation, UI activation) unrelated to clinical content. While some interaction types were conceptually similar (e.g., interacting with UI menus), they appeared in highly abstracted form during the training. In seven of the eight challenges—excluding the orientation task, which assessed indicating a correct direction—performance was measured as successful interactions per time. Participants received immediate performance feedback and repeated tasks if their score fell below the 75th percentile of a pilot cohort. Training duration was capped at 25 min. A full overview of challenges and scoring thresholds is provided in Supplementary Table 1.

Specific IC training was conducted in the STEP-VR emergency simulation environment^18,61^, identical to the assessment setting. Participants followed an audio-guided sequence of seven procedural tasks (e.g., blood sampling, IV access setup), each comprising multiple time steps. Tasks required no medical knowledge but replicated complex interaction patterns. While some low-level interaction mechanics (e.g., object manipulation, navigation, UI activation) overlapped between specific IC training and assessment, none of the high-level clinical tasks or checklist items assessed in the VR exam were rehearsed during training. Accordingly, some tasks involved individual interaction steps that overlapped between specific IC training and assessment (Assessment Items 1, 5, 7 and Supplementary Table 2), while others were entirely distinct from the assessment (Items 2–4, 6, 8, and 9). As in the general IC training, only unsuccessful tasks were repeated. Training was limited to 25 min. Task content and time limits are detailed in Supplementary Table 1.

### VR-based clinical assessment

Participants completed a 9-min VR-based simulation scenario of septic shock due to abscess-forming Crohn’s disease. The scenario focused on implementing the “Sepsis One-Hour Bundle” and initiating guideline-based surgical or interventional measures^62^. Content validity was confirmed by medical educators, and the scenario had been used previously in a VR-based OSCE^63^. Before the assessment, participants received a 5-min orientation to the virtual emergency room and structured case information equivalent to standard OSCE pre-station instructions. The scenario was presented in first-person VR, mirrored to an external screen for supervision and video recording.

### Outcome measures

The primary outcome of the study was clinical performance in the VR-based assessment. Performance was measured with a previously validated OSCE checklist^63^, adapted to the knowledge level of students in the 7th to 10th semester (Supplementary Table 2). The adapted version included nine items, scored either binarily (1 or 0) or ternarily (1, 0.5, or 0). To ensure that the assessment captured genuine clinical competence rather than short-term memory effects, none of the high-level clinical tasks were rehearsed during the IC training. Ratings were based on anonymized video recordings and independently conducted by two blinded raters. All items were equally weighted, and a total performance score was calculated for each participant.

The secondary outcomes were procedural efficiency, cognitive load, as well as self-reported usability barriers and acceptance.

Procedural efficiency was defined as the standardized procedural time (SPT), measured as the interval between the observable initiation and the completion of a procedure. SPT was determined for one previously trained task (intravenous cannulation) and four untrained tasks (blood culture sampling, temperature measurement, abdominal ultrasound, and pneumatic tube use). Since these procedures differed in their inherent time requirements, raw durations were standardized within the sample to *Z*-scores (mean = 0, SD = 1). For untrained tasks, an aggregate score was calculated as the mean of standardized times; lower SPT values indicated higher procedural efficiency.

Cognitive load was assessed both subjectively and objectively. Subjective ratings were obtained using the German version of the unweighted (raw) NASA-TLX^64,65^ at two time points: after 3 min (TLX1, Mental Demand subscale) and immediately after the simulation (TLX2, full scale). Ratings ranged from 0 = lowest to 100 = highest.

Objective data were derived from electrodermal activity (EDA) recordings using the Empatica Embrace Pro wristband. Valid recordings were defined as skin conductance levels between 0.05 and 60 µS and skin temperature between 30 and 40 °C^66^. Data were processed in R (v4.3.1, wearables package^67^) following best-practice standards^68^, with median per minute values used for group-comparisons. A baseline EDA interval was recorded from minute –6 to –4 while participants were already inside the VR assessment environment and received their brief orientation. This setup was chosen to capture VR-related autonomic activation during the baseline without including stress from the clinical scenario.

Finally, self-reported usability barriers and acceptance were evaluated with a post-assessment questionnaire. Items addressed distraction by usability barriers, perceptions of fairness and enjoyment. An open-ended item invited participants to comment on prerequisites for future VR examinations use and responses were subjected to “thematic analysis”^69^.

Participant characteristics and prior experiences were collected, including gender, age and exam performance, operationalized as the mean grade across Internal Medicine, Emergency Medicine, and Anesthesiology according to the German grading system (1 = best, 5 = worst). In addition, participants reported their experience with 3D (computer-based) and VR applications, quantified as the number of hours of use during the past 6 months.

### Software and hardware

General IC training was conducted using the VR Competence App^17^, developed by the Chair for Human-Computer Interaction at the University of Würzburg. Specific training and assessment scenarios were implemented on the STEP-VR platform^61^, an immersive simulation built on a commercial game engine (Unreal Engine 5.1), developed in collaboration with ThreeDee GmbH (Munich, Germany). Interaction logic of both applications was implemented using native controller input and physics-based object handling: object manipulation used a direct-grab paradigm with collision physics and snap-to-socket behavior for connectors (e.g., infusion lines), while UI interactions employed a menu with raycast selection and confirmation buttons, both supported by haptic feedback and contextual audio cues. Locomotion combined smooth walking for short distances with teleportation for longer distances.

The VR setup included an OMEN by HP 17-ck0075ng laptop (Intel Core i7-11800H, NVIDIA GeForce RTX 3070 [8 GB]) and an Oculus Quest III headset with handheld controllers. Applications were rendered at >90 frames per second with high-quality graphic settings.

### Statistical analysis

Normality of residuals was assessed using the Shapiro-Wilk test. For group comparisons, one-way ANOVA was applied when the assumption of normality was met. In cases of deviation, the non-parametric Kruskal–Wallis test was used. Post hoc comparisons of non-normally distributed data were performed using the Mann–Whitney test. For correlation analyses, Pearson’s correlation coefficient was calculated for normally distributed variables, while Spearman’s rank correlation was applied otherwise. To control the false discovery rate arising from multiple comparisons, p-values were adjusted using the Benjamini–Hochberg procedure^70^. Specifically, FDR correction was applied to EDA time-point comparisons, analyses of NASA-TLX subscales, and post-hoc pairwise Mann-Whitney tests following significant Kruskal–Wallis tests of clinical performance (Supplementary Table 5).

To explore interaction effects of covariates, ANCOVA and regression analyses were conducted in R (v.4.5.1 with RStudio v. 2024.04.2). Effect sizes were expressed as Cohen’s *d* with corresponding confidence intervals, with thresholds of 0.2, 0.5, and 0.8 interpreted as small, medium and large effects. Regression equations were reported in the form *Y* = *a* + *bX*, to indicate the magnitude and direction of effects. For cell sizes smaller than five, regression coefficients were not computed^71^. Complete information for key equations can be found in the supplementary material (Supplementary Table 7).

Interrater reliability of checklist scoring was calculated using linear weighted Cohen κ, and group differences in gender distribution were tested using chi-square.

EDA data were processed in R (v4.3.1; RRID:SCR_001905) using a specific wearables package^67^. Median per-minute values were computed for each participant and used for group-level comparisons.

All other analyses were conducted with GraphPad Prism (v10.1.2; RRID:SCR_002798).

Reporting was conducted in accordance with the CONSORT-EHEALTH recommendations (for a completed reporting checklist, see supplementary files)^72^.

### Ethics and data protection

This study was reviewed and approved by the Ethics Committee of the University of Würzburg (Proposal No. 2024-316-ka). Written informed consent was obtained from all participants. All data were processed anonymously and handled in accordance with local data protection regulations. VR interaction data and physiological recordings were stored on encrypted institutional servers and anonymized before analysis. First-person video recordings used for performance rating were de-identified (audio track removed) to ensure participant confidentiality. Participation in the study had no academic consequences for students.

## Supplementary information

Supplementary Information

Consort-ehealthv1.61

Supplementary Data1

## Data Availability

The datasets generated and analyzed in this study are provided in Supplementary Data 1. The video recordings used for performance ratings can be obtained from the authors upon reasonable request.

## Code availability

The VR-Competence App (version 1.1) used for the general IC training was developed by the Chair for Human–Computer Interaction at the University of Würzburg, is not commercially available; inquiries about access for research purposes may be directed to Chair for Human-Computer Interaction. The STEP-VR simulation environment (version 1.0), originally developed by ThreeDee GmbH (Munich, Germany), was used for the specific IC training and assessment procedures in this study. STEP-VR is a licensed simulation platform, and its source code cannot be shared due to intellectual-property and licensing restrictions.
