# Supplementary material for: Immersive competence as a source of bias in virtual reality clinical assessment
Source: NPJ Digit Med. 2026 Mar 9;9:280. doi: 10.1038/s41746-026-02482-z (PMC13049112; doi:10.1038/s41746-026-02482-z)
Supplement: Supplementary file 1 — Supplementary Information [file 41746_2026_2482_MOESM1_ESM.pdf]

Supplementary Table 1. Specifications (content, time limits and target scores) for general and specific IC training.

**General IC training (VR Competence App)**			
**Task No**	**Content**	**Time limit (sec)**	**Target score (75% of pilot cohort)**
1	Controller buttons	60	60%
2	Orientation	60	90%
3	Select items	60	50%
4	Scale items	60	60%
5	Touch user interfaces	60	73%
6	Teleport	60	70%
7	Rotate items	60	60%
8	Raycast for user interfaces	60	70%
**Specific IC training (STEP-VR emergency simulation environment)**			
**Task No**	**Content**	**Time limit* (sec)**	
1	Intravenous cannulation	9	
2	Measure SpO_2	11	
3	Draw blood	16	
4	Administer oral medication	11	
5	Administer intravenous medication	13	
6	Request laboratory results	16	
7	Apply external pacemaker	18	

*The time limits were calibrated to correspond to the 75th percentile of the pilot cohort.

Supplementary Table 2. Candidate assessment form for actions that should be taken during the VR-based assessment for the scenario "Septic shock".

Candidate assessment form for the station "septic shock"			
**Item**	**Result**		
**Monitoring/Diagnostics**			
1 O_2 monitoring attached	Yes		No
2 Blood cultures taken before antibiotic therapy *(partially met: only one pair or sample taken)*	Fully met	Partially met	Not met
3 Arterial blood gas taken	Yes		No
4 Point-of-care ultrasound performed	Yes		No
**Initial Therapy**			
5 Rapid volume replacement initiated *(partially met: flow rate too slow (<500ml/h) or only short IV infusion)*	Fully met	Partially met	Not met
6 Catecholamines (norepinephrine) administered if MAP < 65 mmHg under/after fluid replacement *(partially met: not primarily indicated catecholamine, e.g., adrenaline)*	Fully met	Partially met	Not met
7 Empirical antibiotics administered (1st choice: piperacillin/tazobactam, Meropenem) *(partially met: antibiotic therapy with a 2nd choice drug)*	Fully met	Partially met	Not met
**Further measures and recommendations**			
8 Surgical consultation requested	Yes		no
9 Intensive care unit transfer requested	Yes		no

Supplementary Table 3. Responses to the open-ended question ("The following changes would be necessary for VR-based examinations to be used as a genuine assessment tool in medical education") summarized using thematic analysis.

Theme	n	%	Example quote
Need for extensive prior preparation with the VR environment	71/91	78	"I was frustrated that I was very slow, since I had no prior experience with VR… With regular use, this would certainly become easier"
… practically	66/91	73	"For examinations, one would need to become more familiar with the layout of the simulation environment"
... theoretically	23/91	25	"I would have liked to prepare better and take notes beforehand"
… as part of the curriculum	16/91	18	"In general, I think VR is very useful for practice… as a curricular training, for example as part of the 'emergency medicine weekend', I would find it really good"
Program-related improvements of accessiblity	17/91	19	"The grasping function was sometimes frustrating, otherwise very well implemented"; "Operation was generally easy, but for the medications in the drawer, slightly larger font would be helpful"
Adjustments of examination conditions	11/91	12	"I was a bit frustrated because I could not solve simple tasks due to time pressure"

Supplementary Table 4: Unadjusted and FDR-corrected p-values for clinical performance scores across study groups.

**Group comparison**	**p (uncorrected)**	**p (FDR)**
**I2 vs. I1**	**.005**	**.016**
**I2 vs. CO**	**.015**	**.022**
**I1 vs. CO**	.768	.768

Supplementary Table 5: Descriptive statistics and FDR-adjusted p-values for all NASA-TLX subscales.

Subscale	I1	I2	CO	p (uncorrected)	p (FDR)
**1 Mental demand**	75.81 ± 17.77	80.93 ± 13.90	77.32 ± 10.64	.229	.519
**2 Physical demand**	36.63 ± 24.80	41.97 ± 24.53	23.93 ± 18.26	**.010**	.057
**3 Temporal demand**	76.41 ±13.29	70.67 ± 23.76	75.32 ± 17.93	.912	.912
**4 Performance**	62.56 ± 26.29	65.00 ± 25.15	69.11 ± 27.68	.504	.756
**5 Effort**	71.44 ± 14.28	75.30 ± 15.67	68.43 ± 18.68	.173	.519
**6 Frustration**	65.15 ± 25.80	73.20 ± 18.43	65.86 ± 28.80	.660	.792

Supplementary Table 6: Descriptive statistics and FDR-adjusted p-values for EDA at each time point. Values are reported in µS.

EDA at time point	I1	I2	CO	p (uncorrected)	p (FDR)
**-3**	6.35 ± 4.26	4.16 ± 2.80	1.63 ± 1.77	**0.0002**	**0.003**
**-2**	6.31 ± 4.19	3.84 ± 2.26	1.89 ± 1.92	**0.0008**	**0.006**
**-1**	6.12 ± 4.21	4.05 ± 2.73	1.96 ± 1.90	**0.002**	**0.010**
**0**	4.71 ± 4.16	3.17 ± 2.29	2.32 ± 1.94	0.144	0.198
**1**	5.61 ± 4.16	3.70 ± 2.60	2.94 ± 2.09	0.063	0.126
**2**	5.20 ± 4.40	3.29 ± 2.26	2.94 ± 2.36	0.131	0.198
**3**	5.83 ± 4.47	3.39 ± 2.29	3.15 ± 2.84	**0.040**	0.123
**4**	5.86 ± 4.65	3.44 ± 2.28	3.47 ± 3.01	0.084	0.147
**5**	5.91 ± 4.62	3.53 ± 2.20	3.24 ± 2.39	0.059	0.126
**6**	5.29 ± 4.70	4.09 ± 2.57	2.86 ± 2.48	0.173	0.198
**7**	4.86 ± 2.21	4.10 ± 2.46	3.08 ± 2.58	0.161	0.198
**8**	4.99 ± 2.85	3.99 ± 2.55	3.07 ± 2.44	0.184	0.198
**9**	5.53 ± 2.83	4.10 ± 2.60	2.94 ± 2.58	**0.044**	0.123
**10**	4.71 ± 2.91	3.77 ± 2.19	3.64 ± 3.12	0.567	0.567

Supplementary Table 7: Complete regression results for the key interaction models examining procedural efficiency (H2a) and cognitive load (H2b) as moderators. For each simple slope equation, the regression coefficient (b), standard error (SE), test statistic, confidence intervals (CI), and exact p-value are reported.

*To facilitate readability results are not stratified by the study groups

**Moderator*** / **Clinical performance**							
**Procedural efficiency as a moderator (H2a)**							
**SPT**	**Equation**	**M (*SD*)**	**SE**	**statistic**	**2.5%-CI**	**97.5%-CI**	**p-value**
Low (greater efficiency)	Y = 32.6 - 8.70	32.6 (11.1)	3.32	-2.63	-15.38	-2.03	< .001
High (low efficiency)	Y = 32.6 - 17.3	20.5 (9.67)	4.48	-3.85	-26.28	-8.22	< .001
**Subjective Cognitive load as a moderator (H2b)**							
**NASA-TLX**							
Medium	Y = 7.80 + 18.8	26.6 (9.27)	3.84	9.04	28.52	3.84	< .001
High	Y = 7.80 + 15.1	22.9 (11.0)	3.17	5.61	24.59	3.17	.002
Medium	Y = 11 + 16.0	22.0 (12.3)	1.44	-6.24	38.24	1.44	.155
High	Y = 11 + 13.9	24.7 (10.2)	1.28	-7.93	35.83	1.28	.207
**Objective Cognitive load as a moderator (H2b)**							
**EDA**							
Medium	Y =22.10+6.7	28.8 (4.55)	5.27	1.27	-3.88	17.28	.210
High	Y =22.1-0.10	24.1 (11.0)	11.36	-0.00	-22.88	22.68	.993

Supplementary Data 1: Anonymized datasets generated and analyzed in this study.
(external file: Schaaletal_Supplementary_Data_1.xlsx)
