# Supplementary material for: Immersive competence as a source of bias in virtual reality clinical assessment
Source: NPJ Digit Med. 2026 Mar 9;9:280. doi: 10.1038/s41746-026-02482-z (PMC13049112; doi:10.1038/s41746-026-02482-z)
Supplement: Supplementary file 2 — Consort-ehealthv1.61 [file 41746_2026_2482_MOESM2_ESM.docx]

## Uncovering Immersive Competence as a Hidden Bias in VR-Based Clinical Assessment – A Randomized Controlled Study

## CONSORT-EHEALTH Checklist (V.1.6.1)

**TITLE AND ABSTRACT**

**1a. Identification as a randomized study in the title:**
Yes, the title reads: “Uncovering Immersive Competence as a Hidden Bias in VR-Based Clinical Assessment – A Randomized Controlled Study”.

**1b. Structured summary of trial design, methods, results, and conclusions:**
The abstract includes objectives, participants (n=88), interventions, outcomes, and conclusions.

**INTRODUCTION**

**2a. Scientific background and explanation of rationale:**
The introduction explains the significance of immersive competence (IC) as a potential bias in VR-based assessments.

**2b. Specific objectives or hypotheses:**
Four specific hypotheses (H1–H4) are formulated at the end of the introduction.

**METHODS**

**3. Study Design**

**3a. Description of trial design (such as parallel, factorial) including allocation ratio:**
Single-center, three-arm, randomized controlled trial with a parallel-group design. Participants were randomly allocated in a 1:1:1 ratio to one of three groups: (1) general immersive competence (IC) training, (2) general plus specific IC training, or (3) no structured IC training (control group). Randomization used a computer-generated sequence with fixed block sizes (n=9). Performance outcomes were assessed after the intervention in a single VR-based clinical assessment scenario.

**3b. Important changes to methods after trial commencement (such as eligibility criteria), with reasons:**
No major changes to trial methods were made after commencement.

**4. Participants**

**4a. Eligibility criteria for participants:**
Included advanced medical students (from semester 7 onwards); excluded those with epilepsy or severe simulator sickness.

**4b. Settings and locations where data were collected:**
The study was conducted in the VR lab of University Hospital Würzburg.

**5. Interventions**

**5a. The interventions for each group with sufficient details to allow replication, including how and when they were actually administered:**
Interventions include: I1 – general IC training; I2 – general + specific IC training; CO – no structured training. Each intervention lasted ~25 minutes. Interventions were conducted individually after informed consent and basic VR introduction.

**6. Outcomes**

**6a. Completely defined pre-specified primary and secondary outcome measures, including how and when they were assessed:**
Primary: clinical performance (adapted OSCE checklist); secondary: procedural efficiency (SPT), cognitive load (NASA-TLX and EDA), usability barriers, acceptance.

**6b. Any changes to trial outcomes after the trial commenced:**
No changes were made.

**7. Sample Size**

**7a. How sample size was determined:**
Based on power analysis (α=0.05, power=0.8, effect size f=0.25); minimum 84 participants required; 94 enrolled.

**7b. When applicable, explanation of any interim analyses and stopping guidelines:**
Not applicable.

**8. Randomization**

**8a. Method used to generate the random allocation sequence:**
Computer-generated sequence with fixed block size n=9.

**8b. Type of randomization and any restrictions:**
1:1:1 allocation; fixed block size.

**9. Allocation concealment mechanism**

**Mechanism used to implement the random allocation sequence (such as sequentially numbered containers), describing any steps taken to conceal the sequence until interventions were assigned:**
Allocation was concealed until assignment; participants could not be blinded due to visible training differences; outcome raters were blinded.

**10. Implementation**

**Who generated the random allocation sequence, who enrolled participants, and who assigned participants to interventions:**
Randomization was computer-generated; study personnel enrolled and assigned participants; raters remained blinded.

**11. Blinding**

**11a. Who was blinded after assignment to interventions:**
Outcome raters were blinded; participants were not.

**11b. If relevant, description of the similarity of interventions:**
Not relevant, as participants were not blinded.

**12. Statistical Methods**

**12a. Statistical methods used for primary and secondary outcomes:**
Normality tested via Shapiro-Wilk; group comparisons ANOVA or Kruskal-Wallis; post-hoc Mann-Whitney; correlations via Pearson or Spearman; regression analyses with partial η²; software: R and GraphPad Prism.

**12b. Methods for additional analyses:**
Interaction effects via regression; interaction analysis incorporating procedural efficiency and cognitive load; interrater reliability via Cohen’s κ.

**X26. Ethics and Informed Consent**

**i) Ethics approval:** Approved by Ethics Committee of University of Würzburg (Proposal No. 2024-316-ka).
**ii) Consent:** Written informed consent obtained in person (offline). Participants were informed about study purpose, procedures, risks/benefits, voluntary participation, and withdrawal rights.
**iii) Safety & Security:** Screening for epilepsy or severe simulator sickness, supervised VR sessions in controlled lab, data anonymized, encrypted, and stored securely.

**RESULTS**

**13. Participant Flow**

**13a. Numbers of participants randomized, received treatment, analyzed for primary outcome:**
94 enrolled, 88 analyzed (I1=28, I2=30, CO=30); exclusions due to VR issues, missing videos, incomplete questionnaires. Flow diagram included (Figure 1).

**13b. Losses and exclusions after randomization:**
Six datasets excluded due to technical problems.

**14. Recruitment**

**14a. Dates defining recruitment and follow-up:**
Recruitment from January to August 2025.

**14b. Why the trial ended or was stopped:**
Completed as planned; no early stopping.

**15. Baseline Data**

**Baseline demographic and clinical characteristics for each group:**
Age, gender, exam results, 3D/VR experience reported (Table 1).

**16. Numbers Analyzed**

**Number of participants included in each analysis, whether by original assigned groups:**
n=88; analyzed according to intention-to-treat by assigned groups.

**17. Outcomes and Estimation**

**17a. Results for each primary and secondary outcome, effect size:**
Primary: clinical performance: I2=28.3±10.3%, I1=19.9±10.6%, CO=21.2±10.8%, p=.010, d=0.67; secondary: SPT trained/untrained, cognitive load TLX1/TLX2, usability barriers.

**17b. For binary outcomes, presentation of both absolute and relative effect sizes:**
No binary outcomes reported.

**18. Ancillary Analyses**

**Subgroup or exploratory analyses:**
Correlation between 3D experience and clinical performance and interaction via procedural efficiency and cognitive load were performed for each subgroup.

**19. Harms**

**Important adverse events or unintended effects:**
Technical problems reported in Figure 1; minor simulator complaints not specifically reported.

**22. Interpretation consistent with results, balancing benefits and harms:**

Discussion highlights causal effect of IC, benefits of specific training, limitations, implications for equity in VR assessment.

**DISCUSSION**

**20. Limitations**

Limited by its single-center design, brief single-session IC training, and scenario focus on internal medicine emergencies, which may restrict generalizability. Cognitive load measures were partly subjective or non-specific, and some analyses were exploratory, introducing potential bias and imprecision.

**21. Generalizability**

Conducted in a cohort of medical students; limitations for other populations noted.

**OTHER INFORMATION**

**23. Registration number and name of trial registry:**
Study was a non-clinical educational research trial and was not registered in a clinical trial registry.

**24. Protocol:**
Not reported.

**25. Funding:**
Study received no funding.

**26. Conflicts of interest:**
TM involved in development of STEP-VR software; others declare none.

**27. Access to data:**
Data available as Supplementary Data 1; video recordings on request.
